# Laparoscopic versus open surgery for locally advanced and metastatic gastric cancer complicated with bleeding and/or stenosis: short- and long-term outcomes

**DOI:** 10.1186/s12957-022-02674-3

**Published:** 2022-06-25

**Authors:** Tatyana V. Khorobrykh, Nuriddin M. Abdulkhakimov, Vadim G. Agadzhanov, Davit L. Aghayan, Airazat M. Kazaryan

**Affiliations:** 1 Department of Faculty Surgery №2, I.M.Sechenov First Moscow State Medical University, Moscow, Russia; 2 The Intervention Centre, Oslo University Hospital, Oslo, Norway; 3 Department of Surgery N1, Yerevan State Medical University after M. Heratsi, Yerevan, Armenia; 4 Department of Gastrointestinal Surgery, Østfold Hospital Trust, Grålum, Norway; 5 Department of Surgery, Helse Fonna Hospital Trust, Odda, Norway; 6 Institute for Clinical Medicine, Medical Faculty, University of Oslo, Oslo, Norway

**Keywords:** Laparoscopic surgery, Locally advanced gastric cancer, Stomach cancer, Tumor stenosis, Bleeding from the tumor

## Abstract

**Background:**

Laparoscopic surgery has justified its efficacy in the treatment of early gastric cancer. There are limited data indicating the eligibility of laparoscopic interventions in locally advanced gastric cancer. Publications describing the safety of laparoscopic techniques in the treatment of local and metastatic gastric cancer complicated by bleeding and stenosis are scarce.

**Methods:**

The study included patients with histologically confirmed locally advanced and disseminated gastric cancer and complicated with bleeding and/or stenosis who underwent gastrectomy with vital indications between February 2012 and August 2018. Surgical and oncologic outcomes after laparoscopic surgery (laparoscopic surgery) and open surgery (OS) were compared.

**Results:**

In total, 127 patients (LS, *n* = 52; OS, *n* = 75) were analyzed. Baseline characteristics were similar between the groups. Forty-four total gastrectomies with resection of the abdominal part of the esophagus, 63 distal subtotal (43 Billroth-I and 20 Billroth-II), and 19 proximal gastrectomies were performed. The median duration of surgery was significantly longer in the LS group, 253 min (interquartile range [IQR], 200–295) versus 210 min (IQR, 165–220) (*p* < 0.001), while median intraoperative blood loss in the LS group was significantly less, 180 ml (IQR, 146—214) versus 320 ml (IQR, 290–350), (*p* < 0.001). Early postoperative complications occurred in 35% in the LS group and in 45 % of patients in the OS group (*p* = 0.227). There was no difference in postoperative mortality rates between the groups (3 [6 %] versus 5 (7 %), *p* = 1.00). Median intensive care unit stay and median postoperative hospital stay were significantly shorter after laparoscopy, 2 (IQR, 1–2) versus 4 (IQR, 3–4) days, and 8 (IQR, 7–9) versus 10 (IQR, 8–12) days, both *p* < 0.001. After laparoscopy, patients started adjuvant chemotherapy significantly earlier than those after open surgery, 20 vs. 28 days (*p* < 0.001). However, overall survival rates were similar between the group. Three-year overall survival was 24% in the LS group and 27% in the OS groups.

**Conclusions:**

Despite the technical complexity, in patients with complicated locally advanced and metastatic gastric cancer, laparoscopic gastrectomies were associated with longer operation time, reduced intraoperative blood loss, shorter reconvalescence, and similar morbidity, mortality rates and long-term oncologic outcomes compared to conventional open surgery.

## Introduction

Gastric cancer is the fourth leading cause of cancer death and the fifth most common cancer worldwide. Although there has been a decrease in its incidence and mortality in recent years, over one million new cases were newly diagnosed, and an estimated 769,000 deaths from gastric cancer occurred in 2020 [1]. The prognosis mainly depends on the disease stage at the diagnosis; and since patients are usually diagnosed with locally advanced or metastatic, the prognosis is poor [2–4]. Due to the absence of screening programs in most Western сountries, the late detection of gastric cancer is frequent and is often complicated by bleeding and/or stenosis. Current guidelines do not offer an ultimate standardized approach in the case of development of these complications. The vast majority of these patients receive palliative or symptomatic care, and only in about one fourth of cases, is it possible to perform curative surgery [5–10].

### Bleeding from gastric cancer

The rate of successful endoscopic hemostasis in tumor bleeding, depending on the applied method, can be achieved in 31 to 100% of cases [11, 12], and the incidence of recurrent bleeding reaches 41% [13, 14], which complicates repeated endoscopic interventions and increases mortality [15]. Median overall survival after endoscopic hemostasis is approximately 3–6 months, and mortality (30 days) reaches 22% [13, 16].

The rate of successful hemostasis in transcatheter arterial embolization (TAE) ranges from 40 to 100%, the incidence of recurrent bleeding varies from 41 to 66%, and survival rates and 30-day mortality are 0.9–3.7 months and 25–60%, respectively [17–21].

Palliative radiation therapy at optimal doses is well-tolerated and improves the quality of life of patients with bleeding tumor. The rate of successful hemostasis varies from 50 to 80%, with a median overall survival varying from 2.1 to 5.3 months [22, 23].

Curative and cytoreductive surgeries increase the median survival to 12 months, but the incidents of early postoperative complications can reach 40% [24].

### Malignant gastric obstruction

In the late-period after the application of self-expandable metallic stents (SEMS), 30–50% of patients require repeated interventions due to the complications, [25, 26] and survival of patients does not exceed three months [27].

Symptomatic surgeries (gastrostomy, jejunostomy, gastroenterostomy) in patients with tumor stenosis prolong survival only up to 7 months on average [27, 28]. In case of cytoreductive surgeries, survival rates are noticeably higher, median survival in this group is 10 to 13 months, and 46% of patients may complete adjuvant chemotherapy; at the same time, this parameter in patients after stenting and gastroenterostomy is 22% and 29%, respectively [27, 29].

Laparoscopic surgery has justified its efficacy in the treatment of early gastric cancer [30]. Limited data from Japan (JLSSG-0901), China (CLASS-01), and South Korea (KLASS-02) indicate the eligibility of laparoscopic interventions in patients with locally advanced gastric cancer [31–34]. The subject of the application of laparoscopic technologies on the late stages of gastric cancer remains unresolved, but new publications appear confirming the benefits of minimally invasive surgical interventions for these patients [35]. In this study, we analyze the feasibility and efficacy of laparoscopic gastrectomies in patients with complicated forms of advanced gastric cancer.

## Material and methods

### Study design and participants

We retrospectively assessed outcomes of patients with histologically confirmed locally advanced (the American Joint Committee on Cancer stage T2 and higher) and disseminated gastric cancer and complicated with bleeding and/or stenosis who underwent laparoscopic or open gastrectomy with vital indications between February 2012 and August 2018 in the N.N.Burdenko Departmental Surgery Clinic, affiliated with the Department of Faculty Surgery №1, I.M.Sechenov First Moscow State Medical University, Moscow, Russia. Surgical and oncologic outcomes after laparoscopic surgery (LS) and open-surgery (OS) were analyzed and compared.

Prior to making a decision on surgical treatment strategies, all patients were discussed at a multidisciplinary board meeting. Neoadjuvant chemotherapy was not conducted due to the severe condition of the patients. The choice of open or laparoscopic approach was made depending on the availability of a senior consultant surgeon experienced in the laparoscopic technique.

To establish a clinical diagnosis, before surgery, all patients underwent the following set of diagnostic investigations: esophagogastroduodenoscopy with biopsy, multi-position X-ray of the esophagus, stomach, and duodenum after oral contrast enhancement, contrast-enhanced multi-slice computer tomography of the chest, abdomen, and pelvis.

In case of severe cachexia and anemia before surgery, infusion therapy and additional enteral or parenteral nutrition were administrated.

All patients underwent assessment of the quality of life using the Karnofsky Performance Status (KPS) [36] and the Eastern Cooperative Oncology Group (ECOG) performance status scales [37] before and after (prior starting adjuvant chemotherapy, 18−28 days after surgery) surgery.

### Operative technique

In all laparoscopic interventions, the patients were placed on a supine position on the operating table. Five trocars were used, which were aligned taking into account patients body build and the extent of intervention.

**Fig. 1:**
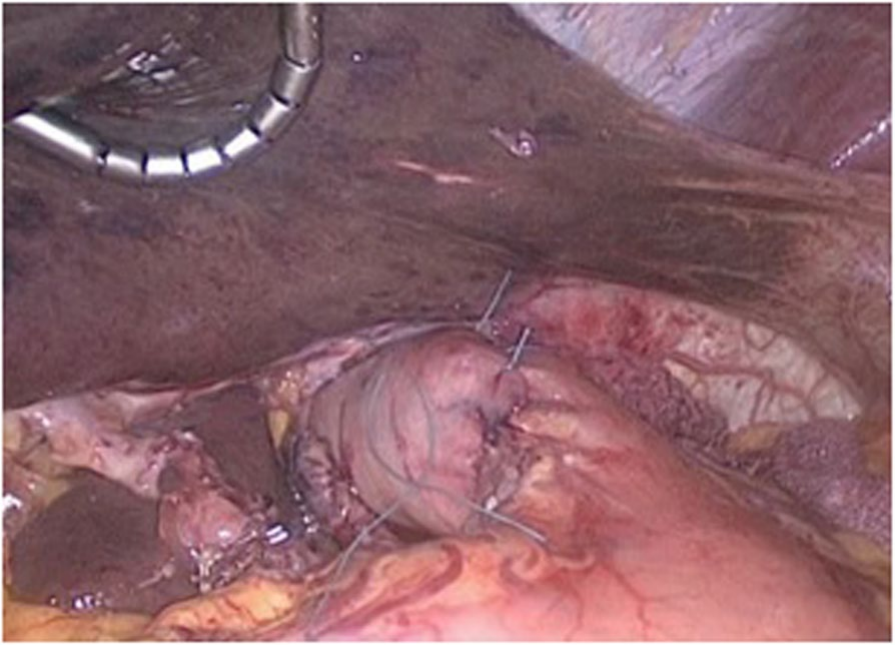
Final result after proximal gastrectomy and esophago-gastrostomy with antireflux cuff (laparoscopic view)

**Fig. 2:**
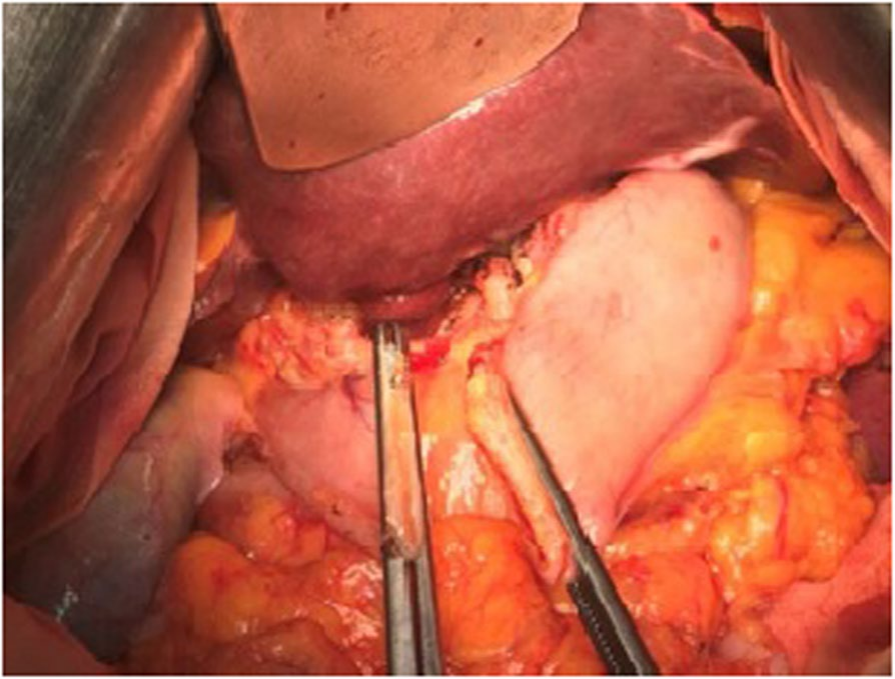
Preparing gastroduodenostomy by B1 (open view)

The extent of surgical intervention depended on the localization of the tumor process. In cases with total and subtotal tumorous affection, and tumors of the upper-third of the stomach, gastrectomy with resection of the abdominal esophagus was performed. Tumors located in the lower-third of the body and in the antral segment of the stomach, distal subtotal resection of the stomach was carried out. In case of the tumor location in the middle-third of the body of the stomach, distal subtotal resection of the stomach was performed if adequate resection was possible, in other cases—gastrectomy was carried out. One to 2 short and posterior gastric arteries were spared for blood supply to the stump. For tumors of the cardia, the method of choice was proximal resection of the stomach with resection of the abdominal and lower-thoracic segments of the esophagus, accompanied by an urgent pathological investigation of the resection margins (Fig. 1). In distal resections, B1 reconstruction (Fig. 2) was preferred, as it is functionally beneficial. The indication for the B2 resection of the stomach was tumor invasion into the pylorus and duodenum or spread to the large curvature closer to the left gastric artery.

**Fig. 3:**
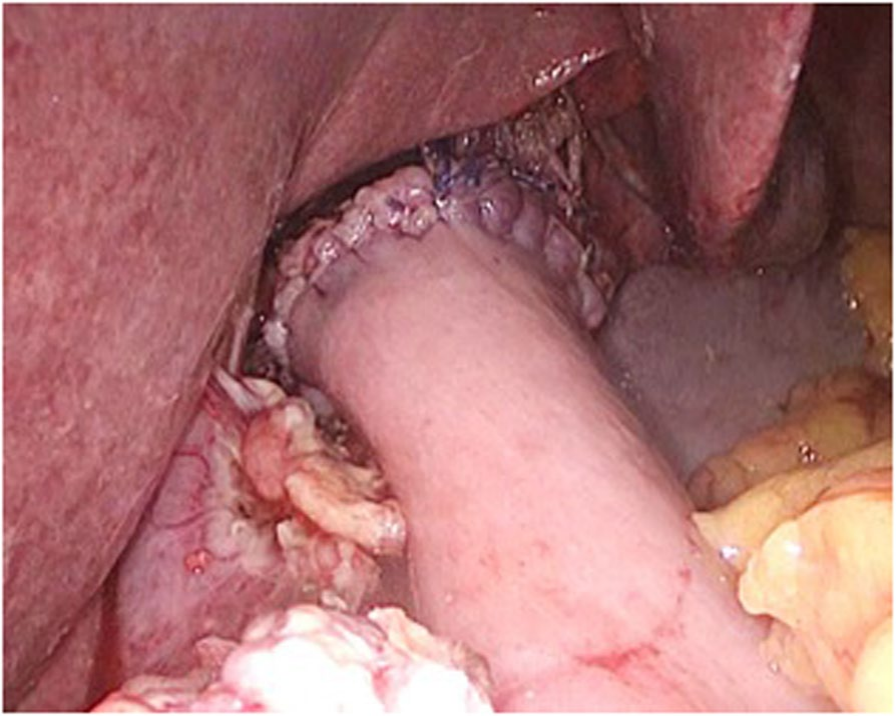
Final result after the imposition of the 1st row of intracorporal esophagojejunostomy (laparoscopic view)

After dissecting the duodenum with a linear stapler during gastrectomy, the staple line area was routinely sutured. Esophago-jejunal anastomosis in the LS group was performed in different ways: in 4 cases—with application of double-row intracorporal manual anastomosis (Fig. 3), in 3 cases—with the use of circular Orvil cross-linking apparatus (head diameter 21 and 25 mm), in other patients it was applied using supramedian minilaparotomy access.

Five to 7-cm-long pararectal minilaparotomy was used to remove the gross specimen and for B1 gastroduodenostomy.

### Early postoperative period

Tube feeding was performed from 1 day via a nasointestinal tube, which was inserted intraoperatively in all patients. On the 3rd–4th day, a control x-ray with a water-soluble contrast was performed, and then the enteral nutrition was started with a gradual increase in volume. As a rule, on the 5th–7th day, the patient independently consumed the desired volume of liquid or strained food [38, 39]. Jejunostomy feeding in this series was not applied.

### Statistics

Procedures were analyzed on intent to treat basis, i.e. cases converted to laparotomy were not excluded from the analyses. The data are presented as median (interquartile range [IQR]) or number (percentage). To compare proportions between groups, the chi-square test or the Fisher exact test were used as appropriate. The Mann-Whitney test was used to compare continuous variables for non-normally distributed variables, while the Student *t* test was used to compare normally distributed variables. The Kaplan-Meier method was applied for survival analyses. Time defined survival values were presented in percentage ± standard error. Log-rank test was applied for comparison of survival between groups. Length of survival was described as median (95% confidence interval [CI]). The reverse Kaplan-Meier method was used to calculate median follow-up of patients for overall survival.

## Results

In total, 127 patients were analyzed. Of them, 52 patients underwent LS and 75 patients had traditional OS. In both groups, the number of male patients was higher, 35 (66%) in the LS and 48 (65%) in the OS group. The median age of patients was 63 years in the open group and 65 in the laparoscopy group (*p* value = 0.074). Physical status of patients before surgery according to ASA classification score was similar between the groups. Other baseline characteristics, such as ECOG score, comorbidities, body mass index, and gastric tumor localization were comparable (Table 1).

**Table 1:** Baseline characteristics

Variables	Laparoscopic surgery (*n* = 52)	Open surgery (*n* = 75)	*P* value
Median age, years (IQR)	65 (58-72)	63 (54-69)	0.074
Gender			
Male	35 (67)	48 (64)	0.710
*ASA status, n (%)*			0.757
ASA I	11 (21)	12 (16)	
ASA II	14 (27)	20 (27)	
ASA III	27 (52)	43 (57)	
*Preoperative ECOG score, n (%)*			0.890
0	11 (21)	18 (24)	
1	32 (62)	43 (57)	
2	9 (17)	14 (19)	
*Comorbidities, n (%)*			
Cardiovascular	49 (94)	71 (95)	1.000
Diabetes type 2	7 (14)	10 (13)	0.983
Peptic ulcer disease	9 (17)	14 (19)	0.845
Chronic obstructive pulmonary disease	4 (8)	5 (7)	1.000
Competing diseases, *n* (%)			0.783
Cholelithiasis	8 (15)	12 (16)	
Colorectal cancer	2 (4)	4 (5)	
Kidney cancer	1 (2)	0 (0)	
Median body mass index kg/m^2^ (IQR)	27.1 (23.4-33.2)	27.1 (24.1-33.7)	0.923
*Tumor localization, n (%)*			1.000
Cardia	14 (27)	19 (25)	
Body	14 (27)	21 (28)	
Antrum	15 (29)	21 (28)	
Subtotal invasion	4 (8)	6 (8)	
Total invasion	5 (9)	8 (11)	
*Cases complicated with, n (%)*			0.688
Bleeding	21 (41)	31 (41)	
Stenosis	22 (42)	35 (47)	
*Proximal*	*10*	*16*	
*Distal*	*12*	*19*	
Both	9 (17)	9 (12)	
*Spread to adjacent organs, n (%)*	28 (54)	48 (64)	0.251
Abdominal esophagus	6 (12)	10 (13)	
Large and small intestine	8 (15)	12 (16)	
Pancreas capsule	4 (8)	6 (8)	
Colon mesentery root	2 (4)	4 (5)	
Diaphragm	0 (0)	3 (4)	
Transverse colon	2 (4)	3 (4)	
Duodenum	6 (11)	10 (13)	
*Metastases (M1), n (%)*	18 (35)	26 (35)	0.995
Liver	2 (4)	3 (4)	
Liver and ovaries	0 (0)	2 (3)	
Liver and peritoneum	1 (2)	2 (3)	
Liver and lungs	2 (4)	1 (1)	
Liver and in the peritoneal fluid	1 (2)	3 (4)	
Ovaries	0 (0)	2 (3)	
Ovaries and lungs	0 (0)	2 (3)	
Lungs	2 (4)	2 (3)	
Peritoneum	5 (10)	5 (7)	
Peritoneum and in the peritoneal fluid	0 (0)	2 (3)	
Peritoneal fluid	5 (10)	2 (3)	

Nine patients in the LS group and 14 patients in the OS group, in addition to the tumor, had ulcerative lesions of the stomach and duodenum (*p* value = 0.845), requiring anti-ulcer therapy.

Forty-four gastrectomies with resection of abdominal esophagus, 44 Billroth-I subtotal resections, 20 Billroth-II subtotal resections, and 19 proximal resections were performed. The extent of gastrectomies were similar between the groups. Combined interventions were significantly higher in the OS group, 32 (62%) versus 59 (79%), *p* value = 0.035. Simultaneous surgeries were carried out in 27 cases. These were equally distributed between the groups (Table 2). Extended lymph node dissection (D2) was performed in 71% patients in LS group and 75 % patients in OS group (*p* value = 0.660). Distribution by stages according to the TNM 8 classification was as follows: IIB–8 patients (6.3%), IIIA–25 (19.7%), IIIB–24 (18.9%), IIIC–26 (20.5%), IV–44 (34.6%). This parameter was similar between the groups (Table 3).

**Table 2:** Performed procedures

Variable	Laparoscopic surgery (*n* = 52)	Open surgery (*n* = 75)	*P* value
Type of gastrectomy, *n* (%)			0.603
Total gastrectomy with resection of the abdominal esophagus	20 (39)	24 (32)	
Distal subtotal gastrectomy	26 (50)	38 (51)	
*Billroth 1*	*19*	*25*	
*Billroth 2*	*7*	*13*	
Proximal gastrectomy with resection of the distal 1/3 of esophagus	6 (11)	13 (17)	
Lymphadenectomy, *n* (%)			0.660
Standard (D1)	15 (29)	19 (25)	
Extended (D2)	37 (71)	56 (75)	
Combined procedures, *n* (%)	32 (62)	59 (79)	0.035
*Resection of the distal 1/3 of esophagus*	*6 (11)*	*10 (13)*	
*Resection of the pancreas capsule*	*4 (8)*	*6 (8)*	
*Resection of the crura of diaphragm*	*–*	*3 (4)*	
*Mesocolon resection*	*2 (4)*	*2 (3)*	
*Colon resection*	*2 (4)*	*3 (4)*	
*Duodenum resection*	*6 (11)*	*8 (11)*	
*Pancreas resection*	*–*	*1 (1)*	
*Splenectomy*	*–*	*3 (4)*	
*Liver resection (mts)*	*6 (11)*	*9 (12)*	
*Ovariectomy (mts)*	*-*	*5 (7)*	
*Peritoneum resection (mts)*	*6 (11)*	*9 (12)*	
Simultaneous procedures, *n* (%)	11 (21)	16 (21)	0.950
*Cholecystectomy*	*8 (15)*	*12 (16)*	
*Right hemicolectomy*	*2 (4)*	*–*	
*Transversum resection*	*–*	*4 (5)*	
*Left kidney resection*	*1 (2)*	*–*

**Table 3:** Perioperative outcomes and histopathology

Variable	Laparoscopic surgery (*n* = 52)	Open surgery (*n* = 75)	*P* value
Median operation time, min (IQR)	253 (200–295)	210 (165–220)	< 0.001
Median blood loss, ml (IQR)	180 (146–214)	320 (290-350)	< 0.001
Conversion to laparotomy, *n* (%)	3 (6)	_	_
Total postoperative complications, *n* (%)	18 (35)	34 (45)	0.227
Sever postoperative complications, *n* (%)	10 (19)	19 (26)	0.397
Postoperative mortality, *n* (%)	3 (6)	5 (7)	1.000
Median ICU stay, days (IQR)	2 (1–2)	4 (3–4)	< 0.001
Median opioid analgesia, days (IQR)	2 (2–3)	4 (3–4)	< 0.001
Median hospital stay, days (IQR)	8 (7–9)	10 (8–12)	< 0.001
Total number of removed lymph nodes, median (IQR)	24 (18–25)	25 (20–26)	0.063
Radicality, *n* (%)			0.688
R0	38 (73)	54 (72)	
R1	10 (19)	12 (16)	
R2	4 (8)	9 (12)	
Type of cancer, *n* (%)			0.904
Adenocarcinoma	40 (77)	57 (76)	
Signet ring cell carcinoma	12 (23)	18 (24)	
*Pathohistological differentiation, n (%)*			0.779
Well differentiated (G1)	8 (16)	15 (20)	
Moderately differentiated (G2)	11 (21)	18 (24)	
Poorly differentiated (G3)	21 (40)	24 (32)	
Undifferentiated (G4)	12 (23)	18 (24)	
*T-stage, n (%)*			0.445
T2	5 (10)	5 (7)	
T3	20 (38)	21 (28)	
T4a	19 (37)	31 (41)	
T4b	8 (15)	18 (24)	
*N-status*			0.774
N0	5 (10)	8 (11)	
N1	12 (23)	15 (20)	
N2	15 (29)	17 (23)	
N3a-N3b	20 (38)	35 (46)	
Cancer stage (TNM 8th), *n* (%)			0.919
IIB	3 (6)	5 (7)	
IIIA	12 (23)	13 (17)	
IIIB	10 (19)	14 (19)	
IIIC	9 (17)	17 (23)	
IV	18 (35)	26 (34)	

Severe complications–higher than Clavien-Dindo grade II

In three (6%) patients, laparoscopy was converted to open surgery. In one case, electric injury of the proper hepatic artery occurred when dissecting a lymph node conglomerate from it. In this case, mini-laparotomy was performed and the injured wall of the artery was sutured. The patient developed an esophageal anastamotic leak postoperatively and died on the 13th postoperative day. In other 2 cases, conversion was performed due to the impossibility of mobilization caused by the massive invasion of the tumor into the root of the mesocolon, and the patients had uneventful postoperative recovery..

Median duration of surgery was significantly longer in the LS group—253 min (IQR, 200–295) versus 210 min (IQR, 165–220) in the OS group (*p* < 0.001), while median intraoperative blood loss in LS group was significantly less and amounted 180 ml (IQR, 146–214) versus 320 ml (IQR, 290–350, *p* < 0.001) (Table 3).

Median number of removed lymph nodes was comparable between the groups (24 (IQR, 18–25) versus 25 (IQR, 20–26), *p* = 0.063). 38 (73%) patients in the LS group and 54 (72%) in the OS group underwent radical (R0) gastrectomy (*p* = 0.688) (Table 3).

Median duration of stay in the intensive care unit after laparoscopic interventions was 2 days (IQR, 1–2), and median duration of the postoperative period was 8 days (IQR, 7–9). In the OS group, these parameters were significantly higher: 4 (IQR, 3–4) and 10 (IQR, 8–12) days, respectively (both *p* < 0.001).

Early postoperative complications in the LS group occurred in 35% and 45% of patients in LS and OS groups, respectively (*p* = 0.227) (Table 3). Detailed characteristics of postoperative complications are presented in Table 4.

**Table 4:** Detailed characteristics of postoperative complications

	Grade (Clavien-Dindo)	Laparoscopic surgery (*n* = 52)	Open surgery (*n* = 75)
Wound infection, *n* (%)	I	2 (4)	4 (5)
Postoperative pancreatitis, *n* (%)	II	3 (6)	4 (5)
Postoperative ileus, *n* (%)	II	1 (2)	2 (3)
Small anastomotic leak (not requiring surgical treatment), *n* (%)	II	2 (4)	5 (7)
Anastomotic stricture, *n* (%)	IIIa	3 (6)	6 (8)
Anastomotic leak (requiring surgical treatment), *n* (%)	IIIb	1 (2)	3 (4)
Bleeding, *n* (%)	IIIb	2 (4)	3 (4)
Pulmonary embolism, *n* (%)	IV	1 (2)	3 (3)
Death of patient, *n* (%)	V	3 (6)	5 (7)

There was no difference in postoperative mortality rates between groups (3 (6 %) in LS and 5 (7%) in OS groups, *p* = 1.00). In the LS group, one patient died on the 2nd postoperative day due to pulmonary embolism, another patient underwent repeated interventions after laparoscopic gastrectomy for esophagoenteroanastomosis dehiscence followed by multi-organ failure. Another patient died on the 8th day after laparoscopic proximal resection of the stomach due to respiratory failure against the background of bilateral polysegmental pneumonia. In the OS group, 3 patients died from pulmonary embolism, 2 patients died on the 10–12th day after surgery due to respiratory and cardiopulmonary failure.

High quality of life before surgery (90–100% according to the Karnofsky scale, 0–1 on the ECOG scale) was observed in 43 (83 %) and 61 (81%) patients in the LS and OS groups, respectively. After surgical treatment, this number increased to 49 (94%) in the LS and 63 (84%) in the OS group. The number of patients who assessed their condition after surgery at 100% on the Karnofsky scale and 0 on the ECOG scale increased 3-fold in LS and 2-fold in OS group (Table 5).

**Table 5:** Assessment of the patients’ quality of life before and after surgery

Variable	Laparoscopic surgery (*n* = 52)		Open surgery (*n* = 75)	
	*Prior surgery*	*After surgery*	*Prior surgery*	*After surgery*
Karnofsky–100%/ECOG–0	11 (21.1%)	30 (57.7%)	18 (24%)	32 (42.7%)
Karnofsky–90%/ECOG–1	32 (61.5%)	19 (36.5%)	43 (57.3%)	31 (41.3%)
Karnofsky–80%/ECOG–2	9 (17.4)	3 (5.8%)	14 (18.7%)	12 (16%)

**Fig. 4:**
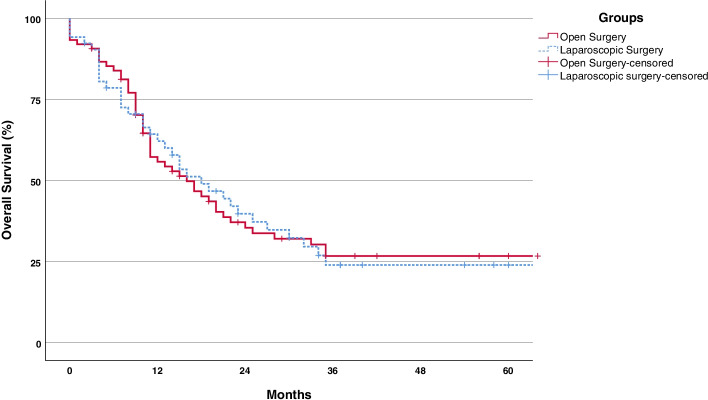
Kaplan-Meier survival curves for overall survival

Median follow-up for overall survival was 56 months (95% CI, 36–76). Overall survival rates were similar between the groups. Median overall survival in the LS and OS groups were 18 (95% CI, 9–23) and 16 months (95% CI, 11–25) (Table 6, Fig. 4).

**Table 6:** Long-term oncologic outcomes

Variable	Laparoscopic surgery (*n* = 52)	Open surgery (*n* = 75)	*P* value
Patients received adj. chemotherapy, *n* (%)	41 (79)	54 (72)	0.382
*Complete*	*33*	*35*	
*Non-complete*	*8*	*19*	
Interval between surgery and adj. chemotherapy, days (IQR)	20 (18–22)	28 (25–30)	< 0.001
Median overall survival, months	18 (10.6–25.4)	16 (9.3–22.6)	0.965
1-year overall survival, % (SE)	62 (± 6.9)	56 (± 5.9)	
2-year overall survival, % (SE)	40 (± 7.2)	35 (± 5.9)	
3-year overall survival, % (SE)	24 (± 5.9)	27 (± 5.6)

**Fig. 5:**
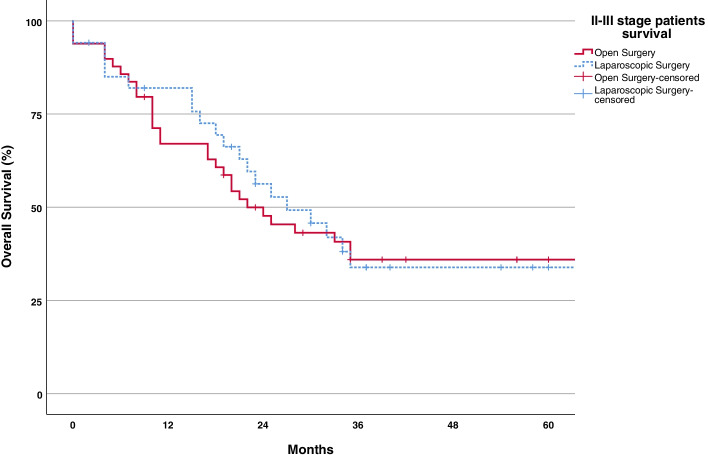
Kaplan-Meier survival curves for overall survival for patients with II–III stage disease LS vs OS

**Fig. 6:**
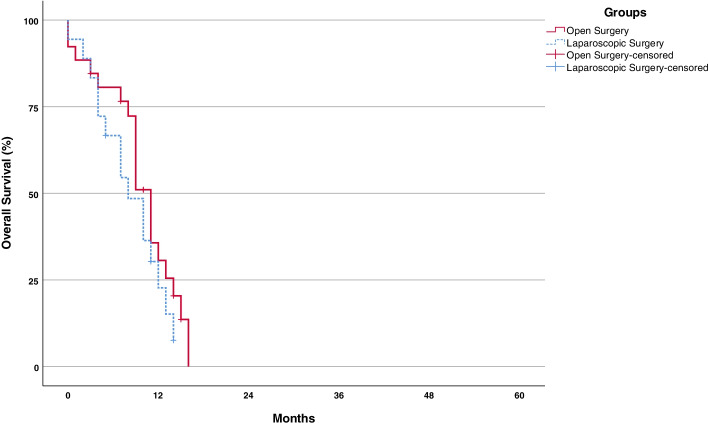
Kaplan-Meier survival curves for overall survival for patients with metastatic disease LS vs OS

After the exclusion of patients with disseminated forms of gastric cancer, the median survival increased to 27 months (95% confidence interval, 16–38) in the LS group (*n* = 34) 22 months (95% CI, 16–38) in the OS group (*p* value = 0.842) (Fig. 5). For those with disseminated disease, the median overall survival was 8 months (95% confidence interval, 4–12) in the LS group (*n* = 26) and 11 months (95 % CI, 9–13) in the OS group (*p* value = 0.274) (Fig. 6).

## Discussion

In this analysis of surgical and oncologic outcomes after laparoscopic and open gastrectomy for patients with locally advanced and metastatic gastric cancer complicated by bleeding and/or stenosis, we found that laparoscopy was associated with significantly less blood loss, shorter ICU and hospital stay, shorter duration of postoperative opioid analgesia, and longer operation time. Other perioperative results including postoperative morbidity and mortality and long-term oncologic outcomes, were similar to the conventional open approach. However, it needs to be stated that the number of patients who required combined procedures due tumor invasion and/or dissemination was higher in the open group.

Among those admitted to the specialized institutions, 80 to 90% of patients, present with locally advanced cancer, metastatic tumor or complications in the form of bleeding or stenosis [40, 41]. In these cases, taking into account the local spread of the process corresponding to stage III–IV, appropriate surgical intervention is an extremely difficult task.

Currently, there are three main strategies in the treatment of complicated gastric cancer: (1) extended curative surgical interventions; (2) cytoreductive and palliative surgeries; (3) conservative tactics based on the endoscopic procedures/fluoroscopic endovascular interventions. To date, curative interventions in case of complications of stage III and IV gastric cancer are challenging and possible in highly selected cases. Even after potentially curative surgery, most of the patients develop early disease recurrence [41].

There are various palliative and symptomatic treatment methods in case of complications of gastric cancer, but they do not provide satisfactory results. The median overall survival of patients after these interventions does not exceed 7 months [13, 16, 20, 22]. However, extended surgical intervention with a radical intention may prolong survival for these patients. The median overall survival after laparoscopic and open surgery in this cohort compiled 18 and 16 months, respectively. In patients with non-disseminated disease median overall survival reached 27 months in the LS group and 22 months in the OS group.

Current clinical guidelines do not offer a standardized approach in case of such complications of gastric cancer as bleeding from the tumor and progressive tumor stenosis.

The latest guidelines (2017) of the Japan Association for Investigation of Gastric Cancer in patients with locally advanced and metastatic gastric cancer with signs of bleeding or obstruction suggest palliative gastrectomy or bypass gastro-jejunostomy depending on the resectability of the primary tumor and surgical risks [6].

American Guidelines (NCCN, 2016) do not recommend active surgical tactics in case of the development of life-threatening complications [7]. In case of acute gastric cancer bleeding, the first options are endoscopic interventions, such as infiltration of the bleeding area, mechanical hemostasis with an endoscopic clamp, argon plasma coagulation, or a combination of various methods. At the same time, it is noted that the efficacy of endoscopic treatment of bleeding in patients with stomach cancer is not sufficiently studied, and the incidence of recurrent bleeding is very high. In case of stomach obstruction with tumor, endoscopic insertion of self-expanding metal stents or percutaneous puncture gastrostomy are performed.

Guidelines of the European Society of Medical Oncology (ESMO, 2016) do not contain any data on the treatment of patients with complicated gastric cancer. There is only casual indication that hypofractionated radiation therapy may be used for reduction of pain, to control bleeding, and in tumor obstruction [8].

In the Korean Clinical Guidelines (March, 2019) [9] based on the results of the discontinued REGATTA study, it is stated that for the treatment of complications (obstruction, bleeding, perforation, etc.), and in order to improve overall survival, palliative surgery is not recommended. However, they do not offer any other treatment options for these patients.

In RUSSCO Practical Guidelines for the Treatment of Gastric Cancer, it is noted that surgical resection of the primary unresectable locally advanced or disseminated/metastatic gastric cancer can be performed in life-threatening complications, which do not resolve with conservative treatment (perforation of the stomach, recurrent bleeding, tumor stenosis, etc.) [42].

Thus, treatment of patients with advanced forms, which include locally advanced (T2-4N0-3M0) and metastatic (M1) gastric cancer is unclear and widely discussed; at the same time, surgical treatment remains the only method that allows both to improve the quality of life of these patients and to increase survival, especially in cases complicated with bleeding, decompensated stenosis, etc.

Laparoscopic surgery has fully justified its efficacy in the treatment of early gastric cancer [43, 44]; however, limited data indicate the eligibility of laparoscopic interventions in local and disseminated processes. Although minimally invasive interventions in locally advanced gastric cancer are technically complex and time-consuming, the data suggest that they may be applied for long-term benefit [45]. In our series, when comparing minimally invasive and open surgeries, there was no significant difference between these two groups with respect to the number of resected lymph nodes, recurrence rate and survival. In addition, minimally invasive technologies provide the best short-term outcomes: low postoperative pain, early activation, faster recovery, and thus shorter intensive care and hospital stay. Considering the fact that the prognosis and the life expectancy of this group of patients is worse, the above mentioned advantages may be essential. Patients after laparoscopic surgeries had significantly higher quality of life, and shorter rehabilitation period allowing to begin the chemotherapy already in early postoperative period. In our series, patients that underwent laparoscopic surgery started their adjuvant chemotherapy significantly earlier than those after open surgery (20 vs 28 days, *p* value < 0.001)

The current analysis has obvious limitations and weaknesses. First, this is a retrospective study with its inherent biases including clear selection bias. Second, the analyzed population and the procedures are heterogeneous. Different types of gastrectomies with different combined and simultaneous procedures were performed. Finally, patients with locally advanced and metastatic disease complicated with bleeding or stenosis referred to palliative treatment were not included in this study, while a comparative analysis with those undergoing gastrectomy could be useful in understanding the role of both laparoscopic and open gastrectomy. All in all, well-designed, multicenter studies, and prospective registries are needed to assess the results of laparoscopic and open gastrectomy in this group of patients.

## Conclusion

The obtained data indicate that despite the technical complexity, in patients with complicated locally advanced and metastatic gastric cancer, laparoscopic gastrectomies were associated with longer operation time, reduced intraoperative blood loss, shorter reconvalescence, and similar morbidity, mortality rates, and long-term oncologic outcomes compared to conventional open surgery. Based on our series, we recommend to apply laparoscopic approach to perform gastrectomy for this group of patients whenever possible

## Data Availability

The datasets used and/or analyzed during the current study are available from the corresponding author on reasonable request.
